# Investigation of Commercial Graphenes

**DOI:** 10.1002/open.202000234

**Published:** 2020-10-19

**Authors:** Stewart F. Parker, Valeri Leich, Jonas Hönig, Peter W. Albers

**Affiliations:** ^1^ ISIS Pulsed Neutron and Muon Source STFC Rutherford Appleton laboratory Chilton OX11 0QX UK; ^2^ School of Chemistry Joseph Black Building University of Glasgow Glasgow G12 8QQ UK; ^3^ Evonik Creavis GmbH Paul-Baumann-Straße 1 45772 Marl Germany; ^4^ Evonik Operations GmbH Kirschenallee 64293 Darmstadt Germany; ^5^ Evonik Technology & Infrastructure GmbH 915 D115 Rodenbacher Chaussee 4 63457 Hanau-Wolfgang Germany

**Keywords:** graphene, inelastic neutron scattering spectroscopy, transmission electron microscopy, X-ray photoelectron spectroscopy

## Abstract

For graphene to achieve its full scientific and commercial potential, reliable mass production of the material on the multi‐tonne scale is essential. We have investigated five samples of graphene obtained from commercial sources that state they can supply the product on the tonne scale per annum. From electron microscopy at the micrometre to the nanometre scale, and neutron vibrational spectroscopy, we find that none of the materials examined were 100 % isolated graphene sheets. In all cases, there was a substantial content of graphite‐like material. The samples exhibited varying oxygen contents, this could be present as carboxylic acid (although other oxygenates, quinones, phenols may also be present) or water. We emphasise that INS spectroscopy is particularly useful for the investigation of inorganic materials that will be used commercially: it provides atomic scale information from macroscopic (10’s of g) amounts of sample, thus ensuring that the results are truly representative.

## Graphical abstract

**Commercial graphenes** from five different sources have been investigated. None of them are present as 100 % isolated sheets, rather they are present as stacks as shown by tilting experiments in the TEM: left, flat on the support, right, tilted 90°. Neutron vibrational spectroscopy of bulk quantities (10’s of g) shows that there are a variety of chemical functionalities and species present in the, nominally, graphene sheets.

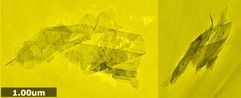

Ever since the first report^1^ of graphene (defined by the International Organization for Standardization (ISO) as “a single layer of carbon atoms^[2]”^), it has been hailed as a “wonder material”.^3^ The applications that have been proposed range from electronic devices4a to catalysis4b to medical applications4c to fillers in composites4d to energy storage4e and, potentially, many others.4f However, to date, most commercial applications are still niche products, such as improved polyurethane foams for heat management4f or in sports goods – tennis racquets,5a golf balls,5b – reinforced by graphene materials.

For graphene to achieve its potential, reliable mass production of the materials on the multi‐tonne scale is essential. There are several methods for the production of graphene.^6^ For applications in electronics, production by chemical vapour deposition can provide large area (cm^2^) defect‐free graphene.^7^ However, this method is expensive and liquid phase exfoliation is generally used for bulk manufacture.^8^ It is natural to ask how successful the manufacturers are at producing genuinely single layer graphene and to this end, there have been two major investigations of the quality of commercial graphene.^9^, ^10^ Both concluded that the overall quality was poor and that most manufacturers were producing largely, or entirely, “nano‐graphite” rather than graphene, as defined by the ISO.

Both of these investigations largely used techniques that only examine very small (nanometres to microns) areas of the sample: light microscopy, atomic force microscopy (AFM), Raman spectroscopy, X‐ray photoelectron spectroscopy (XPS) and electron microscopy (SEM and TEM). While these methods provide detailed atomic level information, there is a concern as to how representative they are of a material that is manufactured on the multi‐kilogram scale. As noted in a commentary^11^ on ref [9]: “there can be no quality without quality control”.

To investigate how this issue may be addressed and to understand the state‐of‐the‐art regarding graphene manufacture, we have sourced a variety of commercial materials from suppliers from Asia‐Pacific and North America, who state that they can supply the product on the tonne scale per annum. We have used a range of techniques to understand the nature of the materials from the macroscale to the atomic scale.

Table 1 lists the samples studied and some of their physical properties (O1s XPS spectra are shown in Figure S1.). It is apparent that there is a considerable variation between them. We note that the theoretical^12^ surface area of graphene is reported as 2630 m^2^ g^−1^, the values in Table 1 are all less than 5 % of this and much closer to that typical of graphite: ∼0.1 m^2^ g^−1^.

**Table 1:** Some properties of the samples studied.

Sample	Production method	H [wt %]	BET [m^2^ g^−1^]	XPS O (at.%)	XPS O1s C−OH/C=O	Number of layers
1	Exfoliation	<0.1	26	2.0	1.225	37
2	Electrochemical exfoliation	0.3	9	4.8	0.75	55
3	Exfoliation	0.2	39	2.8	0.68	48
4	Exfoliation	0.9	83	6.0	0.82	51
5	Exfoliation	0.1	39	2.1	0.42	42

Transmission electron microscopy (TEM) images of two of the samples at two magnifications are shown in Figure 1 (Figures S2 and S3 of the Supplementary Information show all of the samples). Figure 1(a) and (c) shows the samples at low magnification and contrary to the expectation that largely isolated, exfoliated single graphene sheets or entities with a few stacked sheets are mostly present, the carbonaceous particles resemble aggregates and agglomerates (as defined in DIN 53206^13^) and also seen by 3D‐TEM for carbon black.^14^ However, here anisotropic objects of 2D ordering of stacked graphene sheets are under investigation. There are considerable differences between the samples in terms of the size and shape of the agglomerates and the degree of polydispersity. This confirms previous work^9^, ^10^ that the commercial graphene materials are much more complicated than expected from the ideal, “only a single layer of graphene present”.

**Figure 1:**
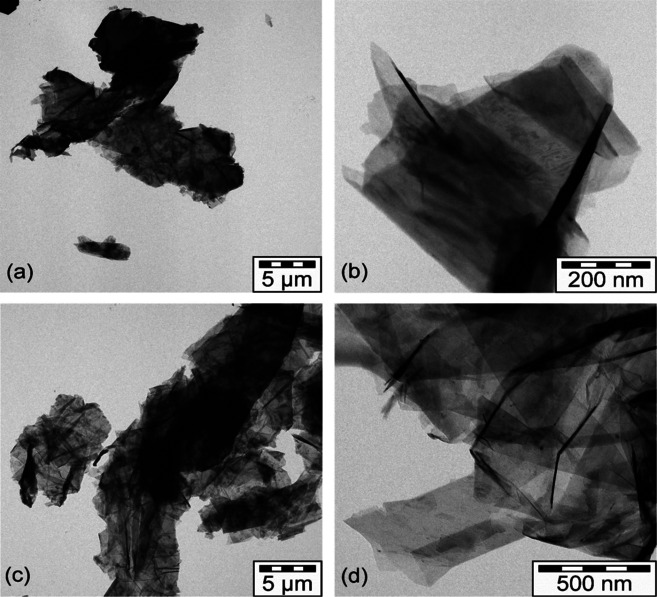
Low resolution (left column) and medium resolution (right column) TEM images of graphene **2**: (a) and (b) and of **5**: (c) and (d).

The images in Figure 1(a) and (c) are of large (microns), thick particles, thus morphological differences over the whole scale from mm → μm → nm complicate the selection of one single typical image for a given grade at some fixed, constant magnification. This emphasisies the difficulty of attempting to characterise the commercial materials by a technique that is highly localised. Unfortunately, most of the analytical techniques, AFM, XPS, Raman spectroscopy, that are commonly used for graphene characterisation suffer from the same limitation.

Within the limitations of the small amount of sample that can be examined by TEM, some features are readily discernible; single layer, few‐layer and multi‐layer graphene and other carbonaceous matter at different proportions and degree of residual aggregation/agglomeration are present. The higher resolution images in Figure 1 (b) and (d) show that there are few isolated sheets, rather that they are highly anisotropic stacks in a “playing cards”‐like arrangement. We emphasise that these images are recorded after intensive liquid phase ultrasonic dispersion which would be expected to break‐up simple clusters. Numerical evaluation of calibrated TEM images, as usually done for commercial carbon blacks, is difficult because of the anisotropy and polydispersity of the carbonaceous objects. Tilting of the samples in the TEM shows how the shape changes with the tilt angle: the anisotropic objects are large but thin, as shown in Figure 2 and in the TEM “movies” in the Supplementary Information (of samples **2**, **3** and **5**). The images of the electrochemically exfoliated **2** suggest the presence of bent sheets, consistent with the presence of corannulene‐type defects.

**Figure 2:**
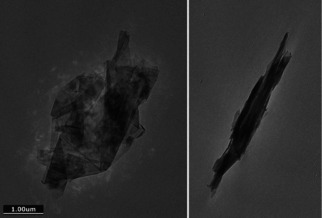
Low resolution images of **3**. Left: lying flat on the sample holder and right: the same sample tilted by 90° in the TEM, showing that it is large, but thin.

The electron microscopy clearly shows distinct physical differences between the samples. To see if this is also true for the chemical structure of the materials, a spectroscopic technique is required. Inelastic neutron scattering (INS) spectroscopy,^15^ is a form of vibrational spectroscopy that has no selection rules and, because neutrons are scattered by the atomic nuclei, the electronic state, be it metallic, semiconducting or insulator is irrelevant. Thus it is ideally suited to highly absorbing, conducting materials such as graphenes.

The measured intensity depends on the scattering cross section of the isotope, this is large for ^1^H and one order of magnitude smaller for any other element (isotope) present. Thus the spectra are dominated by modes involving hydrogen. However, with sufficient sample (as is the case here) non‐hydrogenous materials such as carbon are readily observable. In contrast to all the other techniques that have been used to study commercial graphenes, INS requires relatively large samples: for the work reported here the sample masses were in the range 3–30 g. This highlights the major advantage of INS for the study of graphene: it is a technique that provides detailed chemical information from macroscopic quantities of material.

Figure 3 shows the normalised (to 1 g of sample) INS spectra of the graphene samples, as measured on TOSCA,^16^ an instrument that provides good quality spectra in the 0–2000 cm^−1^ region, but less so at higher energy. Samples **1**, **2** and **4** were also run on a different spectrometer (MAPS) that for reasons explained elsewhere,^17^ allows access to the C−H and O−H stretch region, with better resolution. As found by TEM, there is significant variation between samples that are nominally the same material. Included in the top panel is a reference spectrum of graphite^15^, ^18^ and it can be seen that all of the spectra exhibit features that are characteristic of graphite, these are more pronounced for samples **1**, **3** and **5**. A detailed comparison with sample **1** (which has the lowest hydrogen content, Table 1) is shown in Figure S4 and they are essentially identical. Thus to highlight the spectral features that are *not* due to graphite, the spectrum of sample **1** was subtracted from each of the other spectra. The results are shown in Figure 4.

**Figure 3:**
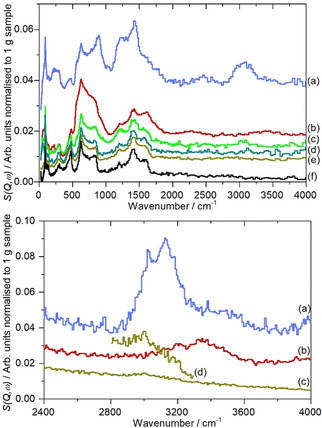
INS spectra of graphene samples. Top panel recorded on TOSCA: (a) sample 4, (b) sample 2, (c) sample 3, (d) sample 5, (e) sample 1 and (f) graphite reference.^18^ Lower panel recorded on MAPS: (a) sample 4, (b) sample 2, (c) sample 1 and (d) sample 1 with ×5 ordinate expansion.

**Figure 4:**
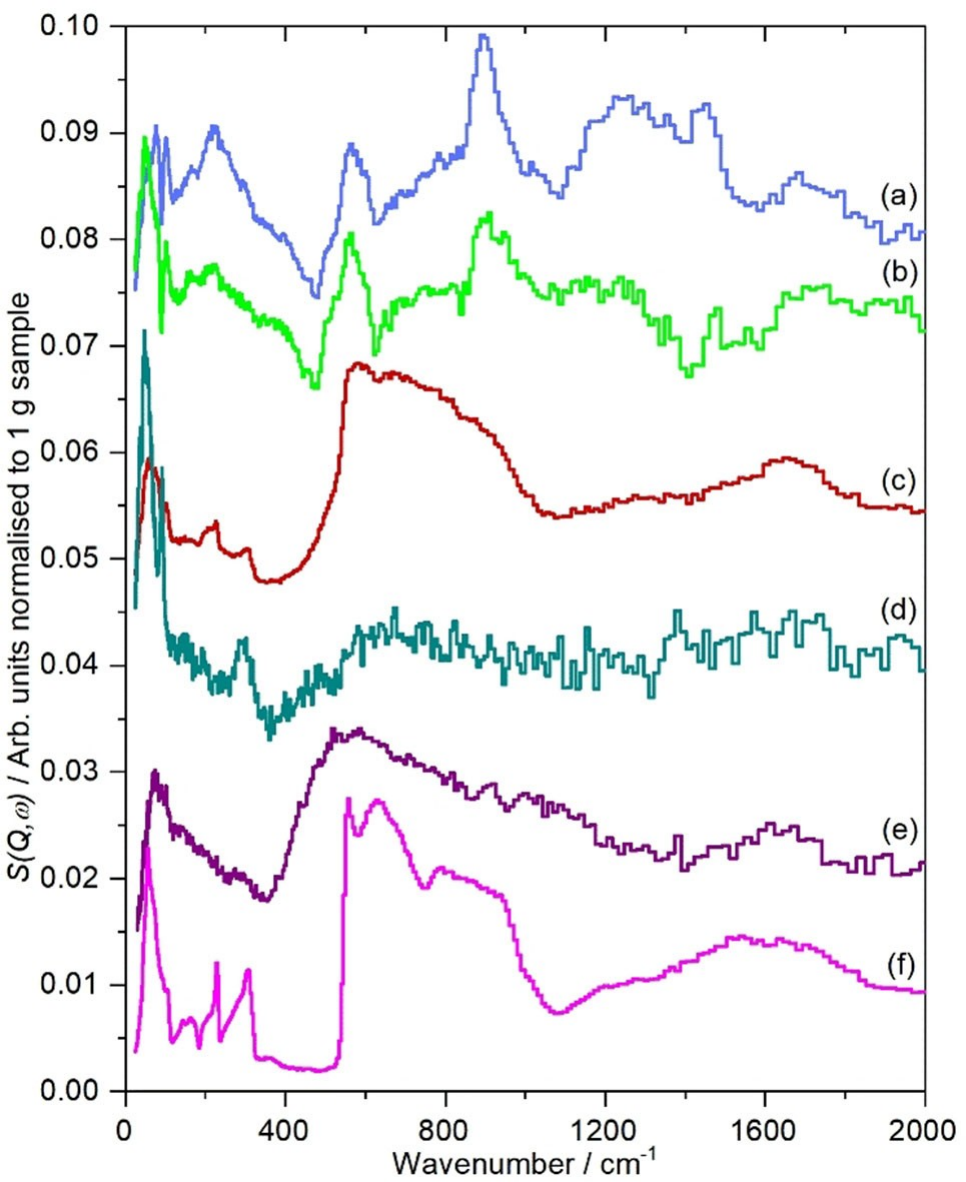
Comparison of INS spectra of graphene samples recorded on TOSCA after subtraction of sample **1**. Some reference ice spectra are also shown. (a) **4**, (b) **3**, (c) **2**, (d) **5**, (e) disordered ice^22^ and (f) ice I_h_. Note the y‐scale only applies to (a)–(d) and that (b) is ×2 and (d) is ×4 ordinate expanded.

The spectra in Figure 4 do not follow any clear trend. Samples **3** and **4** are broadly similar, although the intensity in **4** is *ca*. fourfold greater, consistent with the relative hydrogen concentrations (Table 1) and demonstrating that the features are hydrogen related. Previous work^18^, ^19^ on carbons has shown that the out‐of‐plane bending modes of the edge terminating hydrogen atoms occur at ∼870 cm^−1^ for isolated (H1, no hydrogen neighbours) and at ∼800 and ∼950 cm^−1^ for hydrogen atoms with one hydrogen neighbour (H2). (The corresponding in‐plane bending modes occur at ∼1300 cm^−1^ for both species). It is possible that the peak at 895 cm^−1^ is due to H1 protons, however, the MAPS data (Figure 3, lower panel) shows a complex shape with a tail to high energy, rather than the single peak that would be expected for H1 hydrogen^18^ and as seen for **1** (Figure 4c and d). An alternative assignment would be to carboxylic acid and the XPS results (Table 1) show the presence of a significant oxygen content with a near 1 : 1 ratio of OH and C=O, consistent with the presence of carboxylic acids.

Sample **2** is anomalous in that both the low energy and high energy regions, Figure 3b and 4c, are typical of water. This is very strongly held as overnight evacuation at 120 °C resulted in only a tiny change in mass. Adsorbed water is usually disordered, as seen for water on *e. g*. PdO.H_2_O^20^ and RuO_2_.*x*H_2_O,^21^ Figure 4e and this gives rise to broad, features in both the translational (0–400 cm^−1^) and librational (400–1200 cm^−1^) regions. This is in contrast to the structured features of ice I_h_, Figure 4f. It can be seen that the small amount of water present in **5** is disordered while that in **2** is largely ordered. Sample **2** is the only one that is made by electrochemical exfoliation and it appears from both the electron microscopy (Figure 1(a) and (b)) and the INS spectra that this results in closed voids that contain entrapped water. These must be of reasonable size in order to allow bulk ice I_h_ to form.

INS is an uncommon form of vibrational spectroscopy, so it is useful to compare it with conventional infrared and spectroscopies. Infrared spectroscopy is rarely useful for studies of carbons and these samples illustrate why this is the case. Our attempts to measure the infrared spectra were completely unsuccessful because of the combination of absorption and scattering by the samples so no information was gained. Raman spectroscopy is used extensively for the study of carbons^22^ and graphenes.^9^, ^10^, ^23^, ^24^, ^25^, ^26^ It provides unambiguous characterisation of graphenes in the 1–5 layer range, information about domain size and some information about the quality of the material because the spectra are influenced by the presence of defects. However, Raman spectra of graphenes result from the resonant interaction of the photons with the electronic structure. This results in an enhancement of the Raman cross section by a factor of a million or more and results in exquisite sensitivity. But this has the consequence that any functionality that is not resonantly enhanced is not observed. Thus Raman spectra of graphenes do not show the presence of C−H stretch modes, whereas they are clearly seen by INS. Raman spectroscopy is insensitive to water, thus it would not have seen the trapped water in sample 2 and probably not the (likely) carboxylic acid groups in samples 3 and 4. INS spectroscopy cannot supplant laboratory techniques such as thermal analysis, XPS, infrared or Raman spectroscopies, rather it provides complementary information that is more representative of the bulk of the sample than most other methods, which are generally point sampling techniques.

INS spectroscopy is not a routine laboratory‐based technique: it requires the use of a high intensity neutron source and these are only available at central facilities. There are three such centers currently operational that are capable of the work described here: ISIS (Chilton, UK),^27^ SNS (Oak Ridge, USA)^28^ and J‐PARC (Tokai, Japan).^29^ Access to the facilities is *via* a biannual proposal system and all operate with a free‐at‐the‐point‐of‐use system (*i. e*. beam time is not charged) for work that will appear in the public domain.

In summary, we find that none of the materials examined were 100 % isolated graphene sheets. In all cases, there was a substantial content of graphite‐like material. Based on the surface area and the INS subtractions, we estimate that the fraction of graphene present is probably less than 10 % of the total mass. The samples exhibited varying oxygen contents, this could be present as carboxylic acid (although other oxygenates; quinones, phenols may also be present) or water. For one sample the water was trapped in closed voids and hence inaccessible.

We emphasise that INS spectroscopy is particularly useful for the investigation of inorganic materials that will be used commercially: it provides atomic scale information from macroscopic (10’s of g) amounts of sample, thus ensuring that the results are truly representative. It is clear that for large scale (tonnes) commercial applications, where a uniform, consistent material is required to meet product specifications, the manufacture of industrial quantities of graphene still has some way to go.

## Conflict of interest

The authors declare no conflict of interest.

## Supporting information

As a service to our authors and readers, this journal provides supporting information supplied by the authors. Such materials are peer reviewed and may be re‐organized for online delivery, but are not copy‐edited or typeset. Technical support issues arising from supporting information (other than missing files) should be addressed to the authors.

Supplementary
