# Supplementary material for: Investigation of Commercial Graphenes
Source: ChemistryOpen. 2020 Oct 19;9(10):1060–4. doi: 10.1002/open.202000234 (PMC7569895; doi:10.1002/open.202000234)
Supplement: Supplementary file 1 — Supplementary [file OPEN-9-1060-s001.pdf]

ChemistryOpen

Supporting Information

Investigation of Commercial Graphenes

Stewart F. Parker,* Valeri Leich, Jonas Hönig, and Peter W. Albers

Supporting Information (SI)

Sample preparation.	2
Inelastic neutron scattering spectroscopy (INS).	2
Transmission electron microscopy (TEM).	2
3D-TEM: sample tilt sequences.	3
Surface area and porosity measurements.	3
Table S1. Surface area and porosity of samples **3** - **5**.	3
X-ray photoelectron spectroscopy (XPS).	4
Figure S1. O1s XPS spectra of the graphene samples **1** - **5**.	4
Figure S2. Low resolution (5 μm scale bar) TEM images of graphene samples.	5
Figure S3. Medium resolution (200-500 nm scale bar) TEM images of graphene samples.	6
Figure S4. Comparison of the INS spectrum of graphite and that of sample **1**.	7
References.	8

Sample preparation

The powders were dried in a vacuum oven for 12 h at 105°C to remove any water residues or other volatile matter.

Cylindrical thin walled aluminium cuvettes (0.5 mm wall thickness, internal diameter 4.5 cm and 6.0 cm height) were produced to handle and measure bulk quantities of graphene powders using the full neutron beam profile available (4 x 4 cm). Graphene samples of 10-20 g weight, depending on the pouring density, were filled into the cuvettes and slightly compressed with a piston. The cuvettes were O-ring sealed by a stainless steel top flange and pipe, connected to a welded bellow valve (Swagelok®). After 12 h evacuation using a turbomolecular pump, the sample was heated up to 120°C under vacuum.

Inelastic neutron scattering spectroscopy (INS)

INS spectra were recorded using the MAPS [1] and TOSCA [2] spectrometers at ISIS [3]. On TOSCA the resolution is ~ 1.25% of the energy transfer across the entire energy range, while on MAPS, under the conditions used here, it is ~1.5% of the incident energy at the largest energy transfer and degrades with decreasing energy transfer. Thus TOSCA provides excellent energy resolution at energy transfers < 1200 cm^{-1}, at larger energy transfer MAPS provides better resolution by virtue of the access to low momentum transfer [4]. TOSCA and MAPS are highly complementary and enable the complete range of interest, 0 – 4000 cm^{-1}, to be covered with good resolution. All of the samples were measured on TOSCA, selected ones were also measured on MAPS. On both instruments the samples were cooled to < 20 K and the measurement times were 8 – 12 hours. For the TOSCA spectra, an empty can was subtracted and the data then normalised to 1 g of sample. For the MAPS data, as aluminium has no features in the region of interest above 2000 cm^{-1}, only the sample normalisation was carried out.

Transmission electron microscopy (TEM)

For the TEM investigations the graphene samples were treated by liquid phase dispersion in isopropanol (p.A.)/water (ultrapure) using ultra-sonication with both a focused sonotrode (Hielscher) and an ultrasonic bath (Bandelin). Despite the energy input from the sonication, the materials appeared as partly still compact and partly opaque to the incoming electrons. Very coarse or large particles were also observed, depending on the individual sample. These large particles could not be checked in detail in the TEM and are not included in the images. Note that nm-thin carbon films were used as sample support on the TEM-sample grids.

Two instruments were used. A 100 keV TEM instrument (H-7500 Hitachi, LaB_6 cathode, high contrast mode, ideal to visualize the fine structure of layered systems of low atomic number) enabled comparison of the shape, size and specific micromorphology of the different graphene qualities at the microscale and below. A 200 keV analytical field emission TEM (Jeol 2010 F, with high resolution and ideal for nanospot-analyses by EDX) was used to check morphology and local composition down to the nanoscale and the dimension of graphene sheets as basic structural units which were mostly stacked together as larger aggregates or agglomerates as smallest dispersible units, and to identify other carbonaceous constituents and trace contaminations.

Note that in this work, the terms “agglomerates” and “aggregates” are used as defined in DIN53206 [5] and described previously [6] (and references therein, especially refs. [1-3,7])

3D-TEM: sample tilt sequences

TEM-imaging series generated by changing the sample tilt angle relative to the primary electron beam enables the comparison of the fundamental difference between truly three-dimensional aggregates such as carbon black from *e.g.* the furnace black process or synthetic amorphous silica (from wet phase precipitation or flame hydrolysis processes) (see supplementary material of [6] at: https://onlinelibrary.wiley.com/doi/full/10.1002/crat.201500040) on the one hand and graphene-type material derived from exfoliation processes and various mechanical/chemical post-treatments.

The arrangements of graphene particles, as supported by thin carbon films of TEM sample holders, and shown in Figures S1 and S2 illustrate the differences to carbon blacks, in spite of the intensive liquid phase ultrasonic dispersion:

Agglomerated/aggregated entities of anisotropic material lying flat on the carbon film, carbon particles with lateral sizes up to micrometer dimension (and beyond, see Figure S1). Tilting in the TEM illustrates how the shape changes with the tilt angle: the anisotropic objects are large but thin (see film sample **5**, but also **3** and **2**). The electrochemically exfoliated **2** suggests the presence of bent coherent sheets.

Surface area and porosity measurements

The specific surface area of graphenes was characterized according to DIN ISO 9277 using the Brunauer-Emmett-Teller (BET) method. The total pore volume and pore size distribution were determined from the nitrogen adsorption and desorption isotherms at 77.4 K by the Barrett-Joyner-Halenda (BJH) method. Measurements were performed in duplicate with a Micromeritics ASAP 2420. Analysis of the measurements shows that the material consists mainly of mesoporous material (d = 2-50 nm) and are effective surface areas (according to DIN ISO 9277).

Table S1. Surface are and porosity of samples **3** - **5**.

Sample	BET (m^2/g)	Micropores (d < 2 nm) (ml/g)	Mesopores (d = 2-50 nm) (ml/g)
**3**	39	0.15 (+/- 0.03)	0.87 (+/- 0.06)
**4**	83	0.12 (+/- 0.02)	0.69 (+/- 0.06)
**5**	39	0.12 (+/- 0.02)	0.48 (+/- 0.03)

X-ray Photoelectron Spectroscopy (XPS, ESCA)

To compare the degree of surface oxidation at the edges and defect regions in the basal plane of the graphene sheets and for the presence of other elements integral XPS analyses were performed using a 1 mm spot of surface area and monochromatic AlK_{α} X-rays (ESCALAB 250 Xi).

Directly before an XPS measurement a sample was taken as received from the original shipment container by a micro-spatula and transferred onto a standard powder sample holder of the ESCALAB 250 Xi (Thermo-Fisher) spectrometer system as a loose powder, evacuated at room temperature in a differentially pumped pre-chamber of the spectrometer system (the quality of the environment was checked by a quadrupole mass-spectrometer) and then transferred into the main spectrometer chamber. Under these conditions any possible influence of additional solvent dispersion and energy input by ultra-sonication/tribochemical influence on the original surface chemistry – as received - is avoided. Hence, the sample preparation consisted solely of transferring and pumping down one single sample per measurement from ambient conditions directly from the commercial sample container to oil-free ultrahigh vacuum conditions in a mass-spectrometer-controlled pre-chamber of the XPS spectrometer.

The sp^2 C1s contribution to the XPS signal is strongly asymmetric and the asymmetry parameter and C1s-peak width shows variation, but this fact has often not been taken into account in quantitative XPS analysis [7,8]. Therefore, both the C1s a*nd* O1s signals were checked and evaluated: O1s for the semi-quantitative comparison of the degree of oxidation and C1s as a qualitative check *via* the plasmon-loss feature at about 291 eV as a signature for sp^2 carbon and for the presence of enhanced amounts of carbonyl and carboxyl groups (ca. 286/287 eV and ca. 289 eV). A Shirley-type background subtraction was used.

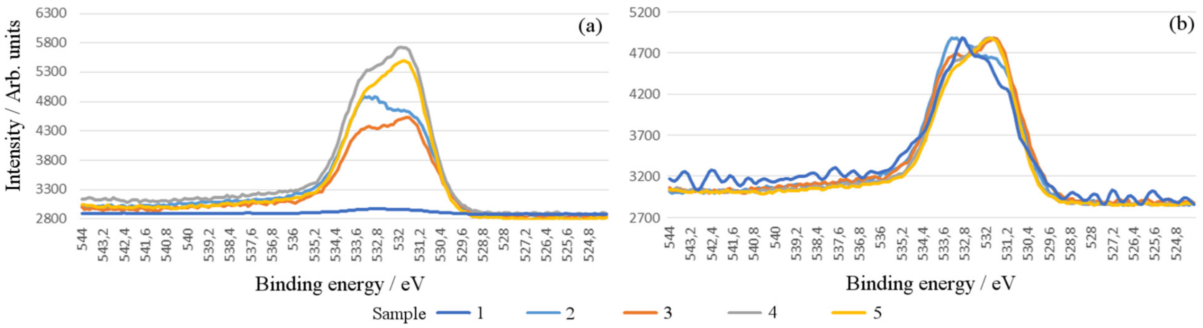

Figure S1. O1s XPS spectra of the graphene samples **1** - **5**. (a) Overlaid spectra and (b) normalised spectra.

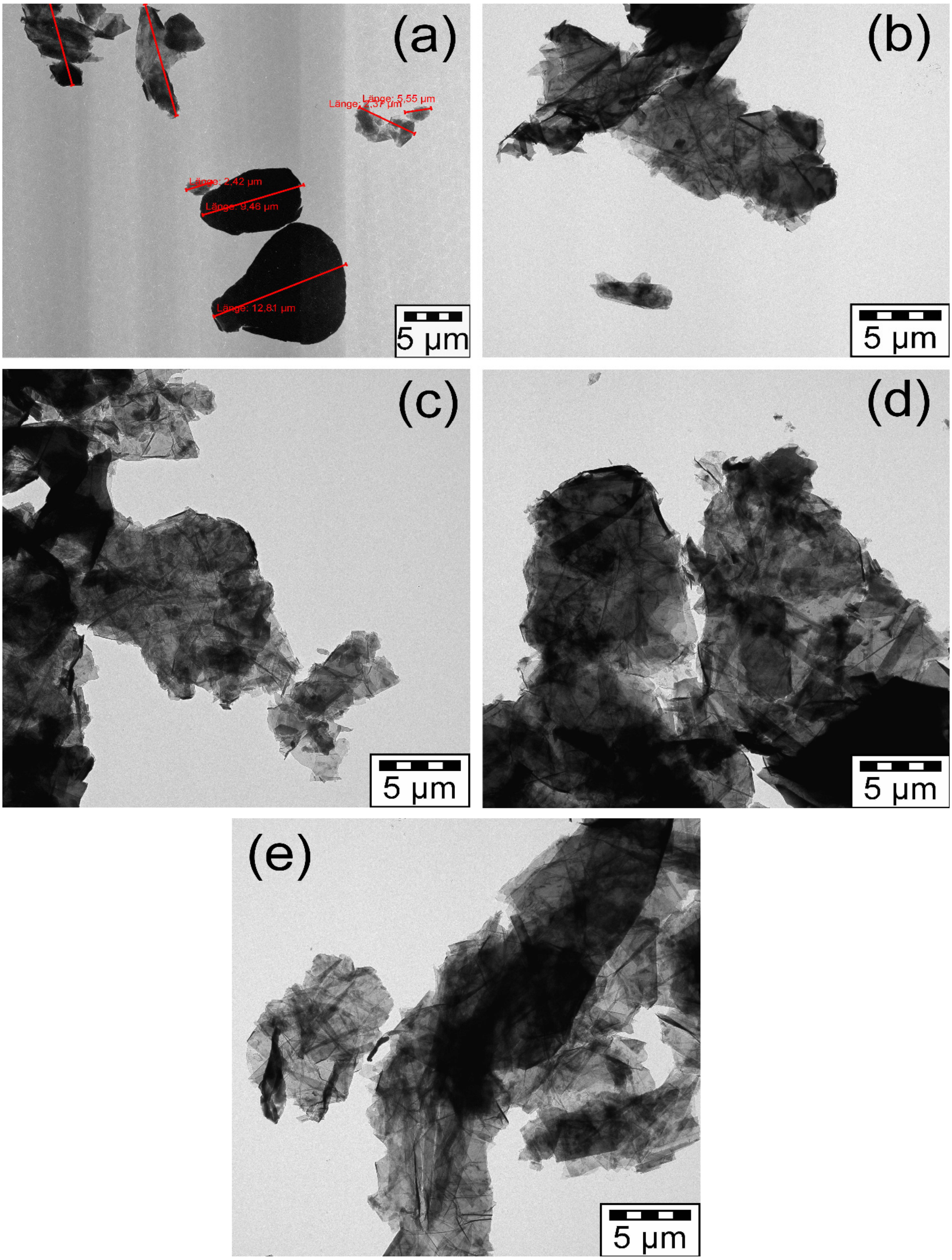

Figure S2. Low resolution (5 µm scale bar) TEM images of graphene samples: (a) **1**, (b) **2**, (c) **3**, (d) **4** and (e) **5**.

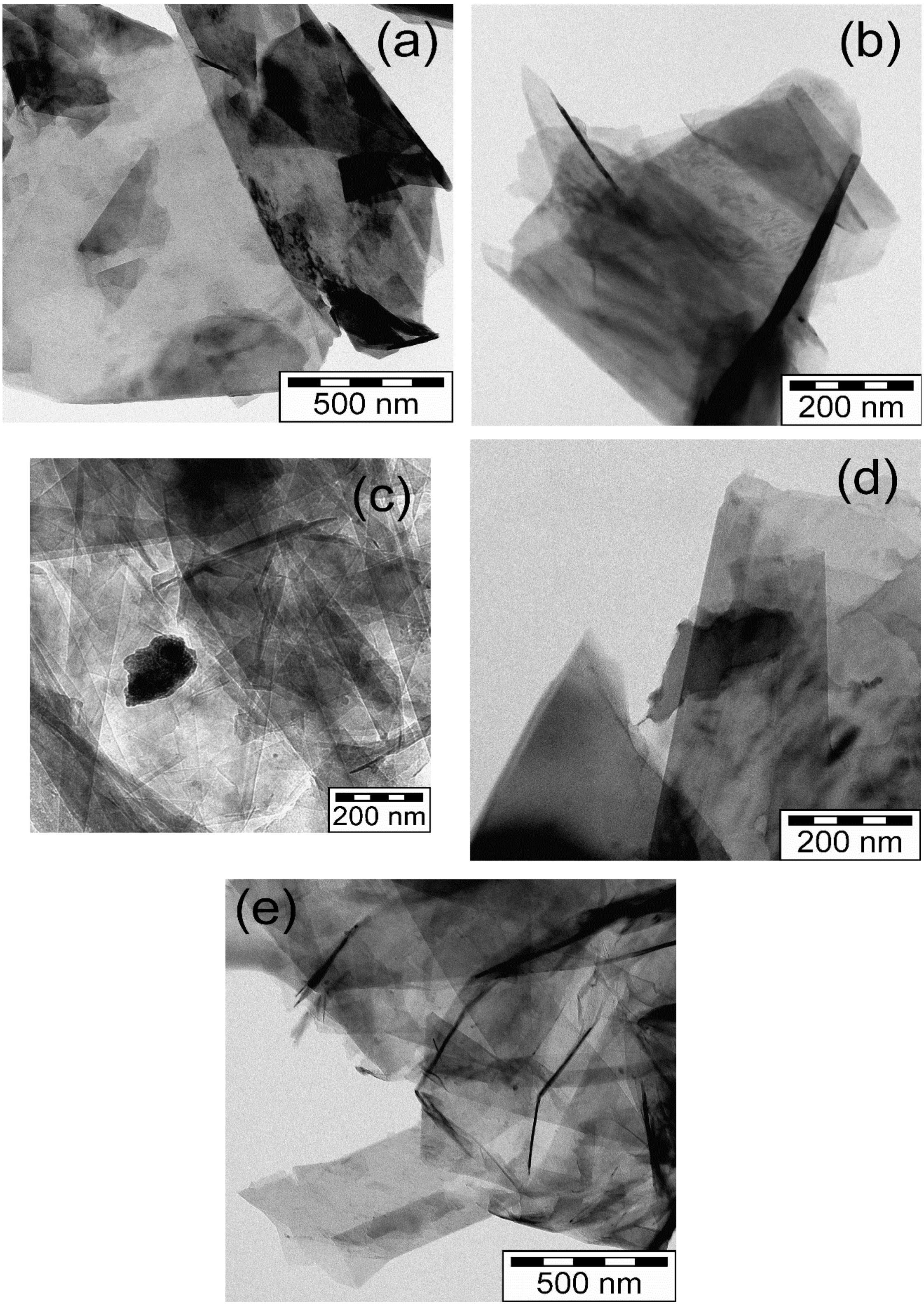

Figure S3. Medium resolution (200-500 nm scale bar) TEM images of graphene samples: (a) **1**, (b) **2**, (c) **3**, (d) **4** and (e) **5**.

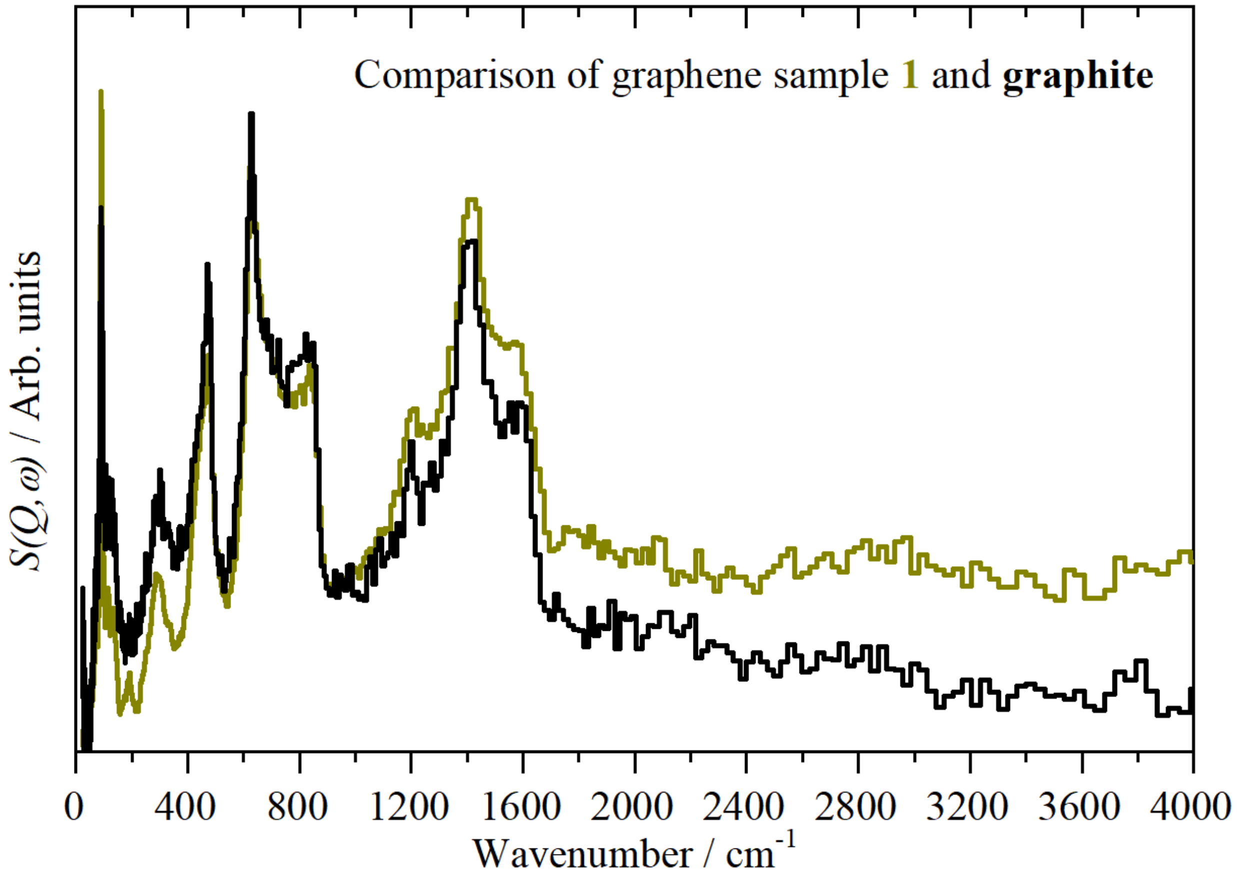

Figure S4. Comparison of the INS spectrum of graphite [9] and that of sample **1**.

References

[1] S. F. Parker, D. Lennon, P. W. Albers, *Appl. Spec.*, **2011**, *65*, 1325-1341.

[2] M. Zanetti, S. Bellissima, L. del Rosso, F. Masi, M. Chowdhury, A. De Bonis, L. Di Fresco, C. Scatigno, J. Armstrong, S. Rudić, S. F. Parker, M. Hartl, D. Colognesi, R. Senesi, C. Andreani, G. Gorini, F. Fernandez-Alonso, *Physica B* **2019**, 562, 107-111.

[3] ISIS Neutron and Muon Source. Available online: http://www.isis.stfc.ac.uk/ (accessed on 20th February 2020).

[4] S. F. Parker, D. Lennon, *J. Phys. Conf. Series* **2016**, *746*, 012066.

[5] DIN 53206-1 Testing of pigments; particle size analysis, basic terms, Deutsches Institut für Normung, **1972**.

[6] P. Albers, M. Maier, M. Reisinger, B. Hannebauer, R, Weinand, *Cryst. Res. Tech.* **2015**, *50*, 846-865.

[7] A. Kovtun, D. Jones, S. Dell'Elce, E. Treossi, A. Liscio, V. Palermo, *Carbon* **2019**, *143*, 268-275.

[8] P. Albers, K. Seibold, G. Prescher, H. Müller, *Appl. Catal. A: General* **1999**, *176*, 135-146 and literature cited therein.

[9] P.W. Albers, W. Weber, K. Möbus, S.D. Wieland and S.F. Parker, *Carbon* **2016**, *109*, 239-245.
